# Biomarkers for diagnosis of sepsis in patients with systemic inflammatory response syndrome: a systematic review and meta-analysis

**DOI:** 10.1186/s40064-016-3591-5

**Published:** 2016-12-12

**Authors:** Yong Liu, Jun-huan Hou, Qing Li, Kui-jun Chen, Shu-Nan Wang, Jian-min Wang

**Affiliations:** 1 Intensive Care Unit, Suining Central Hospital, Deshengxi Road 127, Chuanshan District, Suining, 629000 Sichuan People’s Republic of China; 2 Research Institute of Surgery, Daping Hospital, Third Military Medical University, Chongqing, 400042 People’s Republic of China; 3 State Key Laboratory of Trauma, Burn and Combined Injury, Trauma Center, Chongqing, 400042 People’s Republic of China; 4 Department of Radiology, Institute of Surgery Research, Daping Hospital, Third Military Medical University, Chongqing, People’s Republic of China

**Keywords:** Biomarkers, Sepsis, Systemic inflammatory response syndrome, Diagnosis, Meta-analysis

## Abstract

**Background:**

Sepsis is one of the most common diseases that seriously threaten human health. Although a large number of markers related to sepsis have been reported in the last two decades, the diagnostic accuracy of these biomarkers remains unclear due to the lack of similar baselines among studies. Therefore, we conducted a large systematic review and meta-analysis to evaluate the diagnostic value of biomarkers from studies that included non-infectious systemic inflammatory response syndrome patients as a control group.

**Methods:**

We searched Medline, Embase and the reference lists of identified studies beginning in April 2014. The last retrieval was updated in September 2016.

**Results:**

Ultimately, 86 articles fulfilled the inclusion criteria. Sixty biomarkers and 10,438 subjects entered the final analysis. The areas under the receiver operating characteristic curves for the 7 most common biomarkers, including procalcitonin, C-reactive protein, interleukin 6, soluble triggering receptor expressed on myeloid cells-1, presepsin, lipopolysaccharide binding protein and CD64, were 0.85, 0.77, 0.79, 0.85, 0.88, 0.71 and 0.96, respectively. The remaining 53 biomarkers exhibited obvious variances in diagnostic value and methodological quality.

**Conclusions:**

Although some biomarkers displayed moderate or above moderate diagnostic value for sepsis, the limitations of the methodological quality and sample size may weaken these findings. Currently, we still lack an ideal biomarker to aid in the diagnosis of sepsis. In the future, biomarkers with better diagnostic value as well as a combined diagnosis using multiple biomarkers are expected to solve the challenge of the diagnosis of sepsis.

**Electronic supplementary material:**

The online version of this article (doi:10.1186/s40064-016-3591-5) contains supplementary material, which is available to authorized users.

## Background

Epidemiological surveys indicate that sepsis is the leading cause of non-cardiac death in intensive care units and causes at least 30% of the deaths in patients who are septic (Levy et al. 2010). Along with the aging of the population, the incidence of sepsis shows an obvious increase in countries around the world (Wafaisade et al. 2011; Martin et al. 2003; Angus et al. 2001). An important aspect of improving survival rates in septic patients is early diagnosis, which is helpful to ensure timely treatment and to avoid deterioration of organ function. The classical method of diagnosis is based on signs of an inflammatory response and microbial cultures. However, doctors must wait for several days before getting culture results, and what is worse, negative culture results account for 30–40%. Because microbial cultures have the features of being time-consuming and having a low positive rate as well as being non-specific for systemic inflammatory response syndrome (SIRS), many patients may lose the opportunity of timely and effective treatment. Unlike microbial culture, biomarkers, primarily from the blood, increase in the early stage of the inflammatory response and show different expression between non-infectious inflammation and sepsis. Over the last 20 years, many researchers have been dedicated to finding blood biomarkers for the early diagnosis of infection or sepsis, and they have obtained a substantial number of research results. However, due to the large amounts of experimental data and the inconsistency of the baselines among these studies, it is difficult for medical researchers and workers to make comparisons across various biomarkers or to identify biomarkers with potential diagnostic value. Therefore, we performed a large-scale meta-analysis to summarize potential biomarkers for the differential diagnosis between non-infectious SIRS and sepsis.

## Methods

### Literature search

We conducted the first systematic retrieval from PubMed and Embase in April 2014. The basic retrieval scheme included the following three search keywords: ‘sepsis’, ‘systemic inflammatory response syndrome’ and ‘diagnosis’. Then, we excluded ‘review’, ‘erratum’, ‘editorial’ and ‘letter’ from the retrieval results. In addition, the reference lists of the included original studies and relevant meta-analysis articles were examined for any eligible documents that were missed. The last retrieval was updated in September 2016. The study protocol was approved by the ethics committee affiliated with Daping Hospital and did not require written informed consent from the patients.

### Selection criteria

Articles were included if they evaluated the diagnostic accuracy of biomarkers for distinguishing patients with sepsis from those with non-infectious SIRS. Sepsis was defined as the coexistence of SIRS with infection, according to the diagnostic criteria proposed by the American College of Chest Physicians and the Society of Critical Care Medicine (Bone et al. 1992). We excluded articles that lacked non-infectious SIRS patients as a control group. We also eliminated studies with immunocompromised patients, hematologic patients or pediatric patients. Moreover, articles that could not provide sufficient data to build a 2 × 2 contingency table were likewise excluded.

### Data collection and quality assessment

The data were extracted independently by two reviewers (YL and WX) using a pre-designed Microsoft Excel spreadsheet table that included the categories of methodological quality, methods of biomarker detection, features of the participants and results of diagnostic accuracy. If needed, the authors were contacted for any missing information. We evaluated the quality of the included studies according to the Quality Assessment of Diagnostic Accuracy Studies (QUADAS). Because the analysis of the test results of the biomarkers did not involve clinical data, we omitted item 12 of QUADAS in the quality assessment. Discrepancies between the two reviewers were resolved by discussion with the third author (SHW).

### Data synthesis and statistical analysis

The scheme of the systematic review and meta-analysis was implemented in accordance with the Preferred Reporting Items for Systematic Reviews and Meta-Analyses (PRISMA) statement (Moher et al. 2010). Stata 13.0 software was used to perform the statistical analysis of the pooled data. We used an exact binomial rendition of the bivariate mixed-effects regression model for the synthesis of diagnostic test data (Reitsma et al. 2005). I^2^ statistics were used to reflect the percentage of total variation across articles that were attributable to heterogeneity rather than chance. I^2^ values of 25, 50, and 75% describe the heterogeneity as low, moderate, and high, respectively (Higgins et al. 2003). If heterogeneity existed, and the number of studies was larger than 10, the potential reasons for heterogeneity were identified by meta-regression. Publication bias was evaluated by employing a scatter plot with the inverse of the square root of the effective sample size versus the log diagnostic odds ratio, with a symmetrical funnel shape indicating less publication bias (Deeks et al. 2005).

## Results

**Fig. 1:**
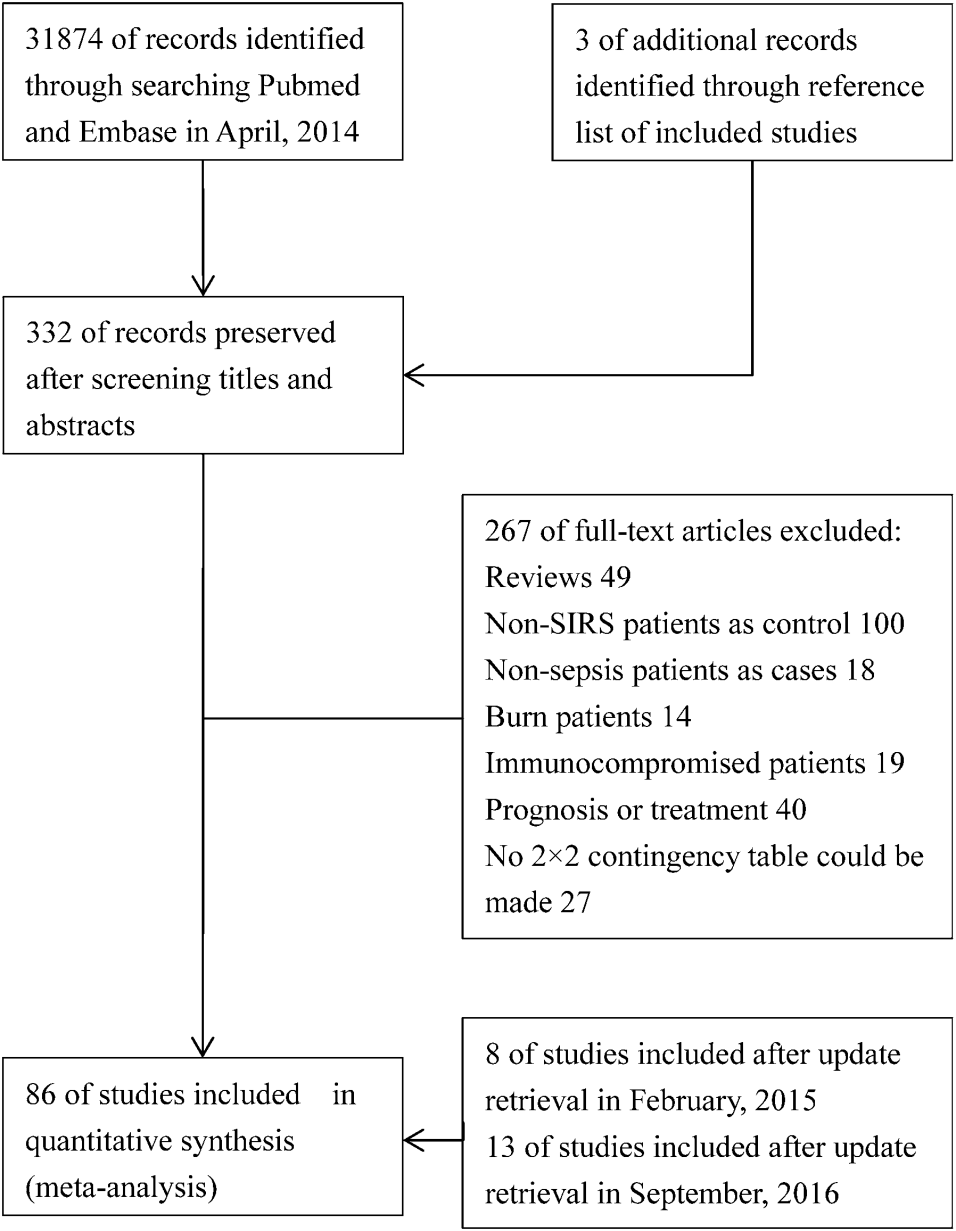
Flow diagram of the study selection

We retrieved articles from the PubMed and EMBASE databases. A total of 31,874 articles remained after duplicates were removed. Three hundred and thirty-two articles were preserved after examining the titles and abstracts. We further excluded 267 articles after reviewing the full content. Sixty-five studies were included in the quantitative synthesis after the first retrieval. Finally, 86 studies were included after two updated searches in February 2015 and September 2016 (Fig. 1) (Abidi et al. 2008; Ahmadinejad et al. 2009; Al-Nawas et al. 1996; Anand et al. 2015; Balc et al. 2003; Barati et al. 2010; Battista et al. 2016; Bell et al. 2003; Beqja-Lika et al. 2013; Carpio et al. 2015; Castelli et al. 2004; Clec’h et al. 2006; de Pablo et al. 2013; Dorizzi et al. 2006; Du et al. 2003; Endo et al. 2012; Farag et al. 2013; Feng et al. 2012; Gaini et al. 2006; Garnacho-Montero et al. 2014; Gerrits et al. 2013; Giamarellos-Bourboulis et al. 2008; Gibot et al. 2004; Godnic et al. 2015; Guven et al. 2002; Han et al. 2016; Harbarth et al. 2001; Hoenigl et al. 2013; Hou et al. 2012, 2016; Hsu et al. 2011; Ishikura et al. 2014; Ivancevic et al. 2008; Jekarl et al. 2013, 2014; Jiang et al. 2015; Kim and Zhang 2012; Kofoed et al. 2007; Latour-Perez et al. 2010; Lewis et al. 2015; Li et al. 2013a; Lin et al. 2015; Matera et al. 2013; Mat-Nor et al. 2016; Mearelli et al. 2014; Meynaar et al. 2011; Miglietta et al. 2015; Miller et al. 1999; Muthiah et al. 2007, Naeini and Montazerolghaem 2006; Oshita et al. 2010; Papadimitriou-Olivgeris et al. 2015; Ratzinger et al. 2013; Reichsoellner et al. 2014; Righi et al. 2014; Rivera-Chavez and Minei 2009; Rogina et al. 2014; Romualdo et al. 2014; Ruiz-Alvarez et al. 2009; Sakr et al. 2008; Scherpereel et al. 2006; Schulte et al. 2011; Selberg et al. 2000; Seok et al. 2012; Shozushima et al. 2011; Sierra et al. 2004; Su et al. 2012, 2013; Sungurtekin et al. 2006; Suprin et al. 2000; Takahashi et al. 2014; Talebi-Taher et al. 2014; Tan et al. 2016; Tian et al. 2014; Tromp et al. 2012; Tsalik et al. 2012; Tsangaris et al. 2009; Tugrul et al. 2002; Ulla et al. 2013; Vaschetto et al. 2008; Vodnik et al. 2013; Wang et al. 2012, 2013; Wanner et al. 2000; Xiao et al. 2015; Yousef et al. 2010). The study by Clec’h et al. reported results separately for medical and surgical patients, and the study by Anand et al. reported results for positive and negative cultures. Furthermore, the study by Lin et al. was divided into a training group and validation group. The results of these three studies were divided into six parts (Anand et al. 2015; Clec’h et al. 2006; Lin et al. 2015).

The main characteristics of the studies are shown in Additional file 1: S1. Altogether, 10,438 patients with non-infectious SIRS or sepsis (including 30,043 test instances) and 60 biomarkers were included in the analysis, of which 18,542 instances (61.72%) indicated sepsis, and 11,501 (38.28%) indicated a SIRS of non-infectious origin. The proportion of sepsis among the studies ranged between 16 and 93% (median 61%).

**Fig. 2:**
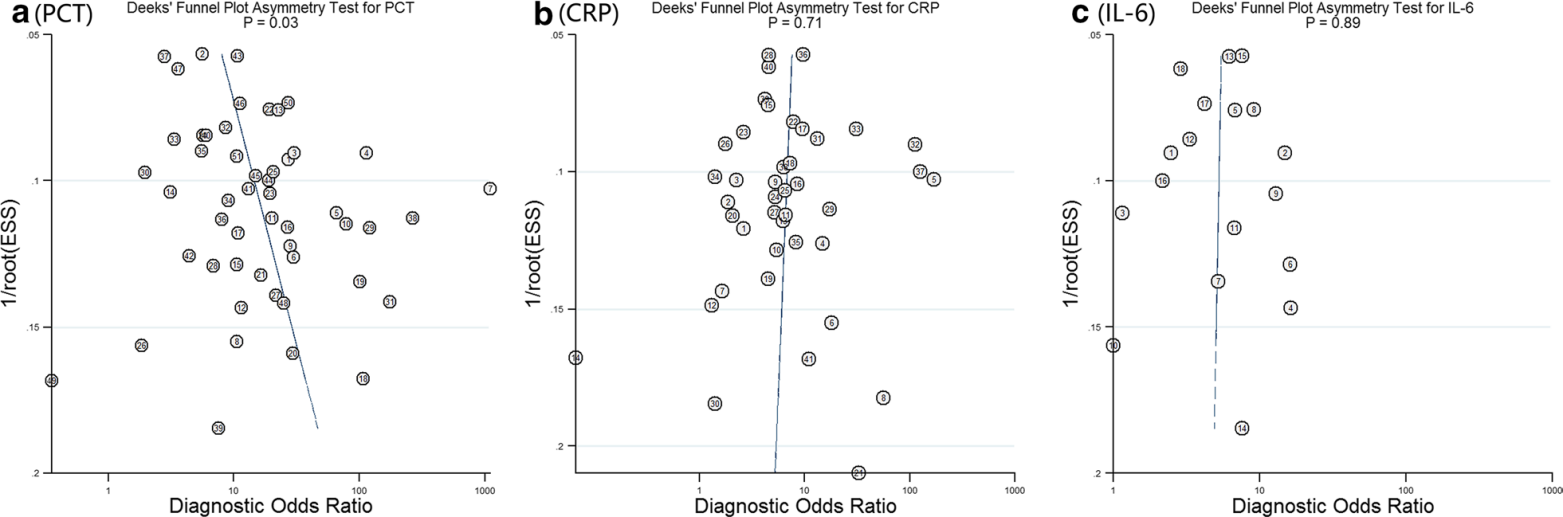
Funnel plots for detection of publication bias of PCT (**a**), CRP (**b**) and IL-6 (**c**)

The methodological quality of the included studies was evaluated according to QUADAS. None of the studies fulfilled all of the items. The included studies fulfilled 766 (69%) of the total 1118 items. The quality was poor for item 10 (index test results blinded), item 11 (reference standard results blinded) and item 13 (uninterpretable results) (Additional file 2: S2). Three biomarkers with more than 10 references, including procalcitonin (PCT), C-reactive protein (CRP) and interleukin 6 (IL-6), were evaluated for publication bias by using Deeks’ regression test of asymmetry (Fig. 2). There was significant publication bias for PCT (P = 0.02) but not for CRP (P = 0.62) and IL-6 (P = 0.70).

Because of there being fewer than 4 references for each, the diagnostic accuracy data of 53 biomarkers could not be pooled by Stata 13.0 software. Thus, we pooled the sensitivity and specificity of 7 biomarkers, including PCT, CRP, IL-6, soluble triggering receptor expressed on myeloid cells-1 (sTREM-1), presepsin (sCD14-ST), lipopolysaccharide binding protein (LBP) and CD64, with 7376, 5654, 3450, 831, 1510, 1136 and 558 participants, respectively, and with the area under the receiver operating characteristic curve (AUC) being 0.85, 0.77, 0.79, 0.85, 0.88, 0.71 and 0.96, respectively (Table 1). The forest plots for the biomarkers are shown in the Additional file 3: S3.

**Table 1:** Research results of biomarkers with at least 4 references

Test	Studies	Cut-off	n	TP	FP	FN	TN	AUC (95% CI)	Sensitivity (95% CI)	Specificity (95% CI)
PCT	59	0.96 (0.5, 1.7) ng/ml^a^	7376	3173	847	1060	2296	0.85 [0.82, 0.88]	0.79 [0.75, 0.83]	0.78 [0.74, 0.81]
CRP	45	84 (38, 140) mg/l	5654	2356	719	1014	1565	0.77 [0.73, 0.81]	0.75 [0.69, 0.79]	0.67 [0.58, 0.74]
IL-6	22	138 (75, 220) pg/ml	3450	1376	403	625	1046	0.79 [0.75, 0.82]	0.72 [0.63, 0.80]	0.73 [0.67, 0.79]
sTREM-1	8	123 (635, 594) pg/ml	831	406	82	126	217	0.85 [0.82, 0.88]	0.78 [0.66, 0.87]	0.78 [0.65, 0.87]
Presepsin	9	600 (415, 647) pg/ml	1510	777	155	168	410	0.88 [0.85, 0.90]	0.84 [0.79, 0.88]	0.77 [0.68, 0.84]
LBP	5	30 (24.35, 32) μg/ml	1136	305	208	191	432	0.71 [0.67, 0.75]	0.62 [0.53, 0.71]	0.70 [0.59, 0.79]
CD64	4	–	558	300	13	76	169	0.96 [0.94, 0.97]	0.87 [0.75, 0.94]	0.93 [0.87, 0.96]

*TP* true positive, *FP* false positive, *FN* false negative, *TN* true negative

^a^Median (25% percentiles, 75% percentiles)

The biomarkers with less than 4 references are displayed in another table (Table 2). Several biomarkers presented high diagnostic values, with AUCs equal to or greater than 0.9 but fewer than 100 participants, including decoy receptor 3 (DcR3), endocan, soluble intercellular adhesion molecule-1 (sICAM-1) and complement 3a (C3a) (with AUCs of 0.96, 0.92, 0.9 and 0.9, respectively).

**Table 2:** The research results for the biomarkers with less than 4 references

Test	References	Cutoff value	N	TP	FP	FN	TN	AUC	Sensitivity	Specificity
*Acute phase protein*										
AGP	Xiao et al. (2015)	1462 mg/l	277	150	8	42	77	0.869	0.782	0.902
MBL	Ruiz-Alvarez et al. (2009)	–	104	49	13	29	13	0.6	0.63	0.5
SAA	Reichsoellner et al. (2014)	289.4 μg/ml	159	80	32	30	17	0.519	0.73	0.35
sPLA2-IIA	Tan et al. (2016)	2.13 μg/l	51	38	2	4	7	–	0.91	0.78
*Biomarkers related to vaosdilation*										
Substance P	Reichsoellner et al. (2014)	0.3 ng/ml	159	62	23	48	26	0.524	0.56	0.53
*Cell marker biomarkers*										
CD64/CD16	Hsu et al. (2011)	–	66	47	2	8	9	0.883	0.855	0.818
CD11C	Lewis et al. (2015)	48.50%	103	67	4	16	16	0.89	0.807	0.8
sCD22	Jiang et al. (2015)	2.3 ng/ml	64	31	6	7	20	–	0.8158	0.7692
sCD163	Feng et al. (2012)	1.49 μg/ml	132	75	2	27	28	0.856	0.74	0.9333
sCD25	Matera et al. (2013)	–	53	25	6	4	18	0.812	0.875	0.75
*Coagulation biomarkers*										
protein C activity	Ishikura et al. (2014)	47%	82	33	7	10	32	–	0.775	0.811
Thrombomodulin	Reichsoellner et al. (2014)	0 ng/ml	159	30	9	80	40	0.543	0.27	0.81
*Cytokine/chemokine biomarkers*										
IFN-r	Jekarl et al. (2014)	45 pg/ml	127	68	16	29	14	0.573	0.702	0.464
IFN-r	Matera et al. (2013)	9 pg/ml	52	13	7	15	17	0.486	0.4545	0.7
IL-1	Jekarl et al. (2014)	30 pg/ml	128	38	8	59	23	0.554	0.394	0.75
IL-10	Jekarl et al. (2014)	40 pg/ml	127	32	1	65	29	0.661	0.329	0.964
IL-10	Matera et al. (2013)	3.05 pg/ml	52	22	5	6	19	0.767	0.7826	0.8
IL-10	Reichsoellner et al. (2014)	1.9 ng/ml	159	25	16	85	33	0.508	0.23	0.67
IL-12	Jekarl et al. (2014)	2 pg/ml	127	18	9	79	21	0.504	0.181	0.714
IL-13	Jekarl et al. (2014)	40 pg/ml	128	85	23	12	8	0.508	0.872	0.25
IL-17	Jekarl et al. (2014)	1.5 pg/ml	127	41	9	56	21	0.586	0.426	0.714
IL-2	Jekarl et al. (2014)	35 pg/ml	127	87	24	10	6	0.534	0.894	0.214
IL-2	Balc et al. (2003)	1288.5 pg/ml	83	22	22	13	26	0.641	0.63	0.55
IL22	Jekarl et al. (2014)	300 pg/ml	127	75	18	22	12	0.542	0.776	0.393
IL-4	Jekarl et al. (2014)	25 pg/ml	127	85	24	12	6	0.516	0.872	0.214
IL-5	Jekarl et al. (2014)	5 pg/ml	127	69	9	28	21	0.714	0.713	0.714
IL-8	Balc et al. (2003)	31.5 pg/ml	83	24	21	11	27	0.663	0.68	0.57
IL-8	Harbarth et al. (2001)	30 ng/ml	78	38	4	22	14	0.71	0.63	0.78
IL-8	Reichsoellner et al. (2014)	507.2 pg/ml	160	50	11	61	38	0.625	0.45	0.77
IL-9	Jekarl et al. (2014)	5 pg/ml	128	83	23	14	8	0.532	0.851	0.25
MIF	Kofoed et al. (2007)	0.81 ng/ml	151	77	29	19	26	0.63	0.8	0.47
Osteopontin	Vaschetto et al. (2008)	1.7 ng/ml	56	19	6	8	23	0.796	0.7	0.79
TNF-α	Balc et al. (2003)	11.5 pg/ml	83	19	16	16	32	0.607	0.55	0.66
TNF-α	Jekarl et al. (2014)	15 pg/ml	128	47	8	50	23	0.598	0.489	0.75
TNF-α	Li et al. (2013a, b)	9.75 pg/ml	52	26	4	12	10	0.796	0.68	0.71
*Receptor biomarkers*										
DcR3	Hou et al. (2012)	2.85 ng/ml	67	23	14	1	29	0.896	0.958	0.674
DcR3	Kim et al. (2012)	3.24 ng/ml	48	24	4	1	19	0.958	0.96	0.826
PLA2-II	Mearelli et al. (2014)	6 ng/ml	80	58	8	2	12	0.851	0.97	0.6
suPAR	Hoenigl et al. (2013)	7.9 ng/ml	132	34	18	21	59	0.726	0.62	0.77
suPAR	Kofoed et al. (2007)	2.7 ng/ml	151	34	18	62	37	0.5	0.35	0.67
suPAR	Reichsoellner et al. (2014)	7.6 ng/ml	160	61	7	50	42	0.66	0.55	0.86
*Vascular endothelial biomarkers*										
Endocan	Scherpereel et al. (2006)	1.2 ng/ml	70	52	0	11	7	0.923	0.825	1
sICAM-1	de Pablo et al. (2013)	904 ng/ml	92	39	2	13	38	0.9	0.743	0.941
*Other biomarkers*										
Ang 2	Mearelli et al. (2014)	3.2 ng/ml	80	49	12	11	8	0.581	0.82	0.4
Biotin	Reichsoellner et al. (2014)	70.4 pg/ml	159	55	9	55	40	0.646	0.5	0.81
C2	Ruiz-Alvarez et al. (2009)	–	104	6	3	72	23	0.5	0.08	0.9
C3	Sungurtekin et al. (2006)	54 mg/dL	99	25	22	16	36	0.566	0.61	0.625
C3a	Selberg et al. (2000)	540 ng/ml	33	19	2	3	9	0.9	0.86	0.8
C4	Sungurtekin et al. (2006)	28 mg/dL	99	32	36	9	22	0.544	0.78	0.382
cf-DNA	Garnacho-Montero et al. (2014)	2850GE/ml	81	41	20	11	9	0.51	0.7931	0.3023
cf-DNA	Hou et al. (2016)	493 pg/ml	67	23	13	1	30	0.856	0.9412	0.7059
Copeptin	Battista et al. (2016)	23.2 pmol/l	90	47	3	17	23	–	0.74	0.87
Cystatin C	Reichsoellner et al. (2014)	2.1 μg/ml	159	55	14	55	35	0.578	0.5	0.71
Delta neutrophil index	Seok et al. (2012)	0.03%	174	93	1	34	46	0.88	–	–
Elastase	Selberg et al. (2000)	91 μg/ml	33	19	10	3	1	0.57	0.86	0.09
eosinophil	Abidi et al. (2008)	–	140	96	4	24	16	0.84	0.8	0.8
Fibronectin	Reichsoellner et al. (2014)	377.4 μg/ml	159	59	15	51	34	0.384	0.54	0.69
Interferon-induced protein 10	Mearelli et al. (2014)	19.5 ng/ml	80	16	0	44	20	0.666	0.27	1
leptin	Farag et al. (2013)	38.05 ng/ml	30	14	0	1	15	–		
Leptin	Yousef et al. (2010)	38 ng/ml	74	36	5	4	29	–	0.912	0.85
miR-143	Han et al. (2016)	15.9 ng/ml	198	81	8	22	87	–	0.786	0.916
miR-146a	Wang et al. (2013)	–	18	6	1	4	7	0.813	0.6	0.875
miR-15a	Wang et al. (2012)	–	198	113	2	53	30	0.858	0.683	0.944
NGAL	Reichsoellner et al. (2014)	82 ng/ml	159	29	2	81	47	0.599	0.26	0.96
Peroxiredoxin4	Schulte et al. (2011)	4.5 U/l	79	32	7	11	29	0.824	–	–
Thrombocytes	Sungurtekin et al. (2006)	–	99	27	17	14	41	0.656	0.659	0.707

Except for CD64, the remaining pooled data of 6 biomarkers showed significant heterogeneity. We conducted a meta-regression analysis for 3 biomarkers (PCT, CRP and IL-6) for which the number of studies was larger than 10. Six factors were analyzed as potential sources of heterogeneity, including sample size, publication year, patient age, patient sex, proportion of patients with sepsis and methodological quality. Although the results of the meta-regression analysis showed that the race that was divided into Caucasian and Asian may be the heterogeneity source for PCT and CRP, the heterogeneity did not disappear in subgroup analysis by race. Therefore, there was no one factor that could satisfactorily explain the heterogeneity source of the three biomarkers.

## Discussion

A total of 60 types of markers were included in our research. Most of the biomarkers had a small number of references. Six biomarkers with the largest number of participants or studies presented a moderate degree of diagnostic value, including PCT, CRP, IL-6, presepsin, LBP and sTREM-1, with AUC values of 0.85, 0.77, 0.79, 0.88, 0.71 and 0.85, respectively. Presepsin and sTREM-1, two popular research biomarkers over the last several years, presented diagnostic values similar to PCT. Several biomarkers with AUCs greater than or equal to 0.9 may be potential biomarkers for sepsis, including CD64, DcR3, endocan, sICAM-1 and C3a. However, the biomarkers with the highest AUCs were described in studies with limited sample sizes and inadequate methodological quality.

Although the reference standard for SIRS and sepsis of the included studies was in accordance with the American College of Chest Physicians and the Society of Critical Care Medicine Consensus Conference, most studies did not provide details that described how the patients were diagnosed with SIRS or sepsis. In some studies, only patients with positive cultures were diagnosed with sepsis, while in other studies, all patients with positive cultures or clinically suspected infections were diagnosed with sepsis. We believe that the cohort being investigated should include different types of patients, such as those with positive cultures and those with clinically confirmed infections. Only in this way can the results of the studies be more representative and have more clinical application value. In addition, we believe studies should exclude the patients whose infection status cannot be confirmed, as these patients may lead to selective bias.

We evaluated the publication bias for three biomarkers, PCT, CRP and IL-6. Among them, the funnel plot of PCT presented publication bias. The PCT funnel plot showed a negative correlation between diagnostic value and sample size. In other words, large sample sizes tended to have a relatively small diagnostic value. Although our meta-analysis only searched two databases, PubMed and Embase, our included references and the results of merged data for PCT were similar to the study by Wacker et al. (2013) who searched 7 databases (pooled sensitivity: 0.79 vs. 0.77; pooled specificity: 0.78 vs. 0.79). Therefore, we believe that one of the major reasons for publication bias in our meta-analysis was more likely the publication of studies with positive or expected results rather than negative results.

Except for CD64, the remaining six biomarkers presented significant heterogeneity. Because the cutoff value for the same biomarker often varied among different studies, the diverse cutoff values often led to the threshold effect as a source of heterogeneity. We used meta-regression analysis to explore the sources of heterogeneity, but no single factor could satisfactorily explain the origins of the heterogeneity, including sample size, publication year, patient age, patient sex, the proportion of patients with sepsis and the methodological quality. Although the heterogeneity among studies was significant, we had stable results for sensitivity analysis. Moreover, the pooled diagnostic test results are consistent with the other meta-analysis results (Wacker et al. 2013; Wu et al. 2012; Li et al. 2013a, b).

Research quality could be an important factor that affected the results. For example, because of limited sample sizes and narrow disease spectra, some studies could not represent the overall state of the patients. In addition, most of the studies did not use blinded methods, which may have resulted in the judgment of sepsis to be affected by the biomarker determination results. These deficiencies may affect the authenticity of results and also lead to heterogeneity among the studies.

In the review by Pierrakos and Vincent (2010), the researchers retrieved a large number of biomarkers related to sepsis and made a detailed classification of them. However, they did not collect all articles in accordance with the inclusion criteria, nor did they quantitatively evaluate the diagnostic value of biomarkers. Two systematic reviews by Wacker et al. (2013) and Wu et al. (2012) evaluated the diagnostic accuracy of two popular biomarkers to differentiate sepsis from SIRS—PCT and sTREM-1, respectively—but they did not include any other biomarkers. In addition, two other systematic reviews by Simon et al. (2004) and Li et al. (2013b) assessed whether biomarkers could diagnose bacterial infection rather than sepsis. Our review included almost all diagnostic trials for the differential diagnosis of septic patients from those with a SIRS of non-infectious origin published before September 2016. Inconsistent control groups from different studies that may result in heterogeneity were ruled out in our analysis, such as those including healthy individuals, infected patients without SIRS, febrile patients without SIRS and immunocompromised patients. We believed the inconsistency of baselines among the control groups would lead to incorrect assessments of the diagnostic value of biomarkers.

The biomarker CD64, a cell surface marker, showed a high value for the differential diagnosis of sepsis and SIRS. However, this test requires flow cytometry and trained technical personnel, which limits its feasibility in clinical applications. Obviously, its high cost means that the promotion of one biomarker will be limited in developing or poor countries. Therefore, we believe that a desirable biomarker for diagnosing sepsis should have the following features: high sensitivity and specificity, elevation in the early phase of the infection, low cost and rapid results.

Sepsis is defined as life-threatening organ dysfunction caused by a serious infection, according to a new international expert consensus (Singer et al. 2016). Therefore, SIRS is not a part of the diagnostic criteria of sepsis according to the new guideline. However, previous studies on sepsis markers still have a certain reference value, such as for PCT, which is still widely used in the clinical setting. Summarizing previous research can provide information for new research and guide the development of new studies.

For the objective assessment of the diagnostic value of septic biomarkers, future trials should compare new putative markers with classical biomarkers such as PCT and CRP in the same trial and follow the Standards for Reporting of Diagnostic Accuracy (Bossuyt et al. 2003). Moreover, medical journals should consider accepting more studies with negative or unintended results to avoid publication bias.

The present meta-analysis shows that plasma PCT, sTREM-1 and presepsin have moderate diagnostic utility in differentiating sepsis from SIRS. Several biomarkers with high AUC values, including CD64, DcR3, endocan, sICAM-1 and C3a, need more studies with larger sample sizes and rigorous methodological designs to confirm the results. Not surprisingly, because sepsis is a non-specific clinical syndrome related to serious microorganism infection and uncontrolled immune responses, it is less likely that one biomarker could satisfactorily differentiate sepsis from SIRS patients. In clinical practice, in addition to the dynamic changes of one septic biomarker, doctors should incorporate biomarkers with medical history, clinical symptoms, physical signs and other tests related to infection when diagnosing sepsis. In the future, biomarkers with better diagnostic value and combined diagnosis with multiple biomarkers are expected to solve the challenge of the diagnosis of sepsis.

## Additional files

**Additional file 1.** Characteristics of the included studies.

**Additional file 2.** The quality assessment of the included studies by QUADAS (Diagnostic Accuracy included in Systematic Reviews).

**Additional file 3.** Forest plots of biomarkers for the diagnosis of sepsis.
