# Supplementary material for: Biomarkers for diagnosis of sepsis in patients with systemic inflammatory response syndrome: a systematic review and meta-analysis
Source: Springerplus. 2016 Dec 12;5(1):2091. doi: 10.1186/s40064-016-3591-5 (PMC5153391; doi:10.1186/s40064-016-3591-5)
Supplement: Supplementary file 1 — Additional file 1. Characteristics of the included studies. [file 40064_2016_3591_MOESM1_ESM.pdf]

S1: Characteristics of the included studies.

Reference	Year	Admission category	Location	n	Age,	Reference	Year	Admission category	Location
Abidi	2008	medical	Morocco	140	43	59	86	CRP	---
Ahmadinejad	2009	medical and surgical	Iran	120	60	49	59	PCT	No information
Al-Nawas	1996	medical	Germany	337	---	---	36	PCT	SSS
Anand	2015 A	---	India	136	51	61	66	PCT, IL-6	SSS
Anand	2015 B	---	India	118	51	62	61	PCT, IL-6	SSS
Balc	2003	medical and surgical	Turkey	83	58	52	42	PCT, CRP, IL-6, IL-8, TNF-α, IL-2	No information
Barati	2010	medical and surgical	Iran	95	58	62	55	CRP, sTREM-1	No information
Battista	2016	---	Italy	90	---	---	71	copeptin, PCT	SSS
Bell	2003	medical and surgical	Australia	83	61	66	75	PCT, CRP	No information
Beqja-Lika	2013	---	Albania	99	44	45	61	PCT, CRP	No information
Carpio	2015	medical and surgical	Peru	123	67	46	93	presepsin	SSS
Castelli	2004	medical and surgical	Italy	49	59	64	69	PCT, CRP	SSS
Clec'h	2006	surgical	France	67	63	66	46	PCT	septic shock
Clec'h	2006	medical	France	76	60	61	47	PCT	septic shock
de Pablo	2013	medical and surgical	Spain	92	62	67	57	sICAM-1	No information
Dorizzi	2006	medical and surgical	Italy	83	66	61	61	PCT	SSS
Du	2003	medical and surgical	China	51	65	61	39	PCT, CRP, IL-6	SSS
Endo	2012	medical and surgical	Japan	185	71	59	62	PCT, IL-6, presepsin	No information
Farag	2013	medical and surgical	Egypt	30	52	33	50	CRP, Leptin	No information
Feng	2012	medical and surgical	China	132	57	64	77	PCT, CRP, sCD163	Sepsis, severe sepsis
Gaini	2006	medical	Denmark	93	63	48	80	PCT, CRP, IL-6, LBP	SSS
Garnacho-Montero	2014	medical and surgical	Spain	160	63	51	64	PCT, CRP, cf-DNA	"severe sepsis,
septic shock"									
Gerrits	2013	---	Netherland	44	72	48	57	CRP, CD64	SSS
Giamarellos	2008	surgical	Greece	69	52		62	sTREM-1	SSS
Gibot	2004	medical	France	76	60	71	62	PCT, CRP, sTREM-1	SSS
Godnic	2015	surgical	Slovenia	47	---	---	85	PCT, CRP, CD64, presepsin	SSS
Guven	2002	---	Turkey	34	56	59	56	PCT, CRP	No information
Han	2016	medical and surgical	China	198	59	69	52	miR-143	SSS
Harbarth	2001	medical and surgical	Switzerland	78	54	73	77	PCT, IL-6, IL-8	SSS

Hoenigl	2013	medical and surgical	Austria	132	67	50	42	suPAR	No information
Hou	2012	surgical	China	67	58		36	DcR3	No information
Hou	2016	medical	China	67	56	61	34	cf-DNA,IL-6 ,PCT	No information
Hsu	2011	respiratory	Taiwan	66	69	77	83	PCT, CD64, CD64/CD16	Severe sepsis, septic shock
Ishikura	2014	medical and surgical	Japan	82	67	54	52	presepsin, Protein C	SSS
Ivancevic	2008	surgical	Serbia	63	55	70	65	PCT	No information
Jekarl	2013	medical and surgical	South Korea	177	52	50	44	PCT, CRP, IL-6	SSS
Jekarl	2014	---	Seoul	127	59	46	76	PCT, CRP, IL-6	SSS
Jiang	2015	surgical	China	64	53	59	59	sCD22, PCT, IL-6	SSS
Kim	2012	medical and surgical	USA	48	55	65	52	DcR3	No information
Kofoed	2007	medical	Denmark	151	56	48	64	PCT, CRP, sTREM-1, SuPAR, MIF	No information
Latour-Perez	2010	medical and surgical	Spain	114	69	61	63	PCT, CRP, sTREM-1	SSS
Lewis	2015	---	UK	103	57	60	81	CD11C, CD64, CRP	No information
Li	2013	surgical	China	52	56	77	73	PCT, IL-6, sTREM-1, TNF-α	SSS
Lin A	2015	medical	China	159	56	79	60	IL-6, PCT	SSS
Lin B	2015	medical	China	74	56	77	51	IL-6, PCT	SSS
Matera	2013	medical and surgical	Italy	52	64	75	54	PCT, CRP, IL-10, sCD25, IFN-γ	No information
Mat-Nor	2016	medical and surgical	Malaysia	239	47	70	69	PCT,IL-6	SSS
Mearelli	2014	medical	Italy	80	83	55	75	PCT, IP-10, PLA2-II, Ang 2	No information
Meynaar	2011	medical and surgical	Netherlands	76	66		42	PCT, CRP, IL-6, LBP	SSS
Miglietta	2015	medical and surgical	Italy	112	62	55	63	PCT, CRP	No information
Miller	1999	surgical	USA	35	43	71	83	CRP	No information
Muthiah	2007	medical and surgical	Australia	130	64	51	29	PCT	Severe sepsis, septic shock
Naeini	2006	medical and surgical	Iran	50	56	64	50	PCT	SSS
Oshita	2010	---	Japan	168	74	68	67	PCT,CRP	No information
Papadimitriou	2015	medical and surgical	Spain	66	50	53	44	CD64, CRP	No information
Ratzinger	2013	medical and surgical	Austria	298	58	58	72	LBP	No information
Reichsoellner	2014	medical	Austria	159	66	51	69	PCT	No information
Righi	2014	medical and surgical	Italy	93	59	77	66	CRP, CD64	SSS
Rivera-Chavez	2009	surgical	USA	93	35	75	60	sTREM-1	SSS
Rogina	2014	---	Slovenia	88	64	50	47	PCT, CRP, CD64	SSS
Romualdo	2014	medical and surgical	Spain	226	68	58	16	PCT, CRP, presepsin	No information
Ruiz-Alvarez	2009	medical and surgical	Spain	103	67	73	76	PCT, CRP, C2, MBL	SSS

Sakr	2008	surgical	Germany	327	63	63	36	PCT, CRP, IL-6, LBP	SSS
Scherpereel	2006	medical and surgical	Switzerland	70	54	59	90	Endocan	SSS
Schulte	2011	medical and surgical	Switzerland	78	60	51	54	PCT, CRP, Peroxiredoxin4	SSS
Selberg	2000	medical	Germany	33	48	61	67	PCT, CRP, IL-6, C3a, Elastase	SSS
Seok	2012	medical	Korea	165	63	55	73	CRP	SSS
Shozushima	2011	medical and surgical	Japan	41	62	61	71	presepsin	SSS
Sierra	2004	medical and surgical	Spain	125	49	84	56	CRP	SSS
Su	2012	medical and surgical	China	144	55	64	58	CRP	No information
Su	2013	medical and surgical	China	130	57	63	77	PCT, sTREM-1	SSS
Sungurtekin	2006	medical and surgical	Turkey	99	59		41	CRP, C3, C4	No information
Suprin	2000	medical and surgical	France	95	57	65	79	PCT, CRP	SSS
Takahashi	2014	medical and surgical	Japan	456	77	58	79	PCT, CRP, IL-6，Presepsin	SSS
Talebi-Taher	2014	---	Iran	100	76	49	50	PCT, CRP, IL-6	No information
Tan	2016	medical and surgical	Malaysia	51	54	54	82	CD64, sPLA2-IIA	No information
Tian	2014	neurological	China	104	54	64	54	PCT, CRP	SSS
Tromp	2012	medical	Netherland	342	59	57	16	PCT, CRP, IL-6, LBP	No information
Tsalik	2012	medical and surgical	USA	336	52	52	74	PCT, CRP, IL-6	SSS
Tsangaris	2009	medical and surgical	Greece	50	63	76	54	PCT	SSS
Tugrul	2002	medical and surgical	Turkey	85	45	46	88	PCT, CRP	SSS
Ulla	2013	medical and surgical	Italy	189	64	61	56	PCT, presepsin	SSS
Vaschetto	2008	unclear	Italy	56	50	77	48	osteopontin	Severe sepsis, septic shock
Vodnik	2013	surgical	Serbia	60	54.4	58	50	Presepsin	No information
Wang	2012	medical and surgical	China	198	60	70	84	miR-15a	No information
Wang	2013	---	China	18	---	---	56	miR-146a	No information
Wanner	2000	surgical	Switzerland.	133	40	74	34	PCT	No information
Xiao	2015	medical and surgical	China	277	50	49	69	AGP,CRP,PCT	SSS
Yousef	2010	medical and surgical	Egypt	74	44	53	54	leptin	SSS

SSS: Sepsis, severe sepsis and septic shock; NA, not available.
