# Supplementary material for: Biomarkers for diagnosis of sepsis in patients with systemic inflammatory response syndrome: a systematic review and meta-analysis
Source: Springerplus. 2016 Dec 12;5(1):2091. doi: 10.1186/s40064-016-3591-5 (PMC5153391; doi:10.1186/s40064-016-3591-5)
Supplement: Supplementary file 2 — Additional file 2. The quality assessment of the included studies by QUADAS (Diagnostic Accuracy included in Systematic Reviews). [file 40064_2016_3591_MOESM2_ESM.pdf]

S2: The quality assessment of the included studies by QUADAS (Diagnostic Accuracy included in Systematic Reviews).

Reference	year	1. Patient spectrum	2. Selection criteria	3. Reference standard	4. Disease progression bias	5. Partial verification bias	6. Differential verification bias	7. Incorporation	8. Description of index test	9. Description of reference standard	10. Test review bias	11. Diagnostic review bias	13. Uninterpretable results	14. Withdrawals
Abidi	2008	no	yes	yes	yes	yes	yes	yes	yes	yes	unclear	yes	unclear	unclear
Ahmadinejad	2009	yes	yes	yes	yes	yes	yes	yes	yes	yes	yes	yes	unclear	yes
Al-Nawas	1996	unclear	yes	yes	yes	yes	yes	yes	yes	yes	unclear	unclear	unclear	unclear
Anand	2015	unclear	yes	yes	yes	yes	yes	yes	yes	yes	unclear	unclear	no	yes
Balc	2003	yes	yes	yes	yes	yes	yes	yes	yes	yes	unclear	unclear	unclear	unclear
Barati	2010	yes	yes	yes	yes	yes	yes	yes	yes	yes	unclear	yes	unclear	yes
Battista	2016	unclear	no	yes	yes	yes	yes	yes	yes	yes	unclear	unclear	yes	yes
Bell	2003	yes	yes	yes	yes	yes	yes	yes	yes	yes	unclear	unclear	unclear	unclear
Beqja-Lika	2013	unclear	yes	yes	yes	yes	yes	yes	yes	yes	unclear	unclear	unclear	unclear
Carpio	2015	yes	yes	yes	yes	yes	yes	yes	yes	yes	unclear	unclear	unclear	yes
Castelli	2004	no	yes	yes	yes	yes	yes	yes	yes	yes	yes	yes	unclear	unclear
Clec'h	2006	yes	yes	yes	yes	yes	yes	yes	yes	yes	unclear	unclear	unclear	yes
de Pablo	2013	yes	yes	yes	yes	yes	yes	yes	yes	yes	unclear	yes	unclear	unclear
Dorizzi	2006	yes	yes	yes	yes	yes	yes	yes	yes	yes	unclear	unclear	unclear	yes
Du	2003	unclear	yes	yes	yes	yes	yes	yes	yes	yes	unclear	yes	unclear	no

Endo	2012	yes	yes	yes	yes	yes	yes	yes	yes	yes	unclear	unclear	unclear	no
Farag	2013	unclear	yes	yes	yes	yes	yes	yes	yes	yes	unclear	unclear	unclear	unclear
Feng	2012	yes	yes	yes	yes	yes	yes	yes	yes	yes	unclear	unclear	unclear	yes
Gaini	2006	no	yes	yes	yes	yes	yes	yes	yes	yes	unclear	unclear	unclear	unclear
Garnacho-Montero	2014	yes	yes	yes	yes	yes	yes	yes	yes	yes	unclear	unclear	unclear	unclear
Gerrits	2013	no	yes	yes	yes	yes	yes	yes	yes	yes	unclear	unclear	unclear	no
Giamarellos-Bourboulis	2008	no	yes	yes	yes	yes	yes	yes	yes	yes	unclear	unclear	unclear	yes
Gibot	2004	yes	yes	yes	yes	yes	yes	yes	yes	yes	unclear	yes	yes	yes
Godnic	2015	no	yes	yes	yes	yes	yes	yes	yes	yes	no	no	yes	yes
Guven	2002	unclear	yes	yes	yes	yes	yes	yes	yes	yes	unclear	yes	unclear	unclear
Han	2016	yes	yes	yes	yes	yes	yes	yes	yes	yes	unclear	unclear	yes	yes
Harbarth	2001	yes	yes	yes	yes	yes	yes	yes	yes	yes	yes	yes	unclear	yes
Hoenigl	2013	unclear	yes	yes	yes	yes	yes	yes	yes	yes	unclear	unclear	unclear	unclear
Hou	2012	no	yes	yes	unclear	yes	yes	yes	yes	unclear	unclear	unclear	unclear	yes
Hou	2016	no	yes	yes	yes	yes	yes	yes	yes	yes	no	no	yes	yes
Hsu	2011	no	yes	yes	yes	yes	yes	yes	yes	yes	yes	yes	unclear	yes
Ishikura	2014	yes	yes	yes	yes	yes	yes	yes	yes	yes	unclear	unclear	unclear	yes
Ivancevic	2008	no	yes	yes	yes	yes	yes	yes	yes	yes	unclear	unclear	unclear	yes
Jekarl	2013	yes	yes	yes	yes	yes	yes	yes	yes	yes	unclear	unclear	unclear	yes
Jekarl	2014	yes	yes	yes	yes	yes	yes	yes	yes	yes	unclear	unclear	no	yes
Jiang	2015	no	unclear	yes	yes	yes	yes	yes	yes	yes	unclear	unclear	no	yes
Kim	2012	yes	yes	yes	unclear	yes	yes	yes	yes	unclear	unclear	unclear	unclear	yes
Kofoed	2007	no	yes	yes	yes	yes	yes	yes	yes	yes	unclear	yes	unclear	yes
Latour-Perez	2010	yes	yes	yes	yes	yes	yes	yes	yes	yes	unclear	yes	unclear	unclear

Lewis	2015	unclear	yes	yes	yes	yes	yes	yes	yes	yes	unclear	yes	yes	yes
Li	2013	yes	yes	yes	yes	yes	yes	yes	yes	yes	unclear	unclear	unclear	yes
Lin	2015	no	yes	yes	no	yes	yes	yes	yes	yes	unclear	unclear	no	yes
Matera	2013	no	yes	yes	yes	yes	yes	yes	yes	yes	unclear	unclear	unclear	unclear
Mat-Nor	2016	yes	yes	yes	yes	yes	yes	yes	yes	yes	unclear	yes	unclear	yes
Mearelli	2014	yes	yes	yes	yes	yes	yes	yes	yes	yes	unclear	unclear	unclear	yes
Meynaar	2011	yes	yes	yes	yes	yes	yes	yes	yes	yes	unclear	yes	unclear	yes
Miglietta	2015	unclear	yes	yes	yes	yes	yes	yes	yes	yes	no	no	yes	yes
Miller	1999	no	yes	yes	yes	yes	yes	yes	yes	yes	unclear	unclear	unclear	unclear
Muthiah	2007	yes	yes	yes	yes	yes	yes	yes	yes	yes	yes	yes	unclear	yes
Naeini	2006	unclear	yes	yes	yes	yes	yes	yes	yes	yes	unclear	unclear	unclear	no
Oshita	2010	unclear	yes	yes	yes	yes	yes	yes	yes	yes	unclear	unclear	unclear	no
Papadimitriou	2015	no	unclear	unclear	yes	yes	yes	yes	yes	yes	unclear	unclear	no	yes
Ratzinger	2013	yes	yes	yes	yes	yes	yes	yes	yes	yes	unclear	unclear	unclear	no
Reichsoellner	2014	no	yes	unclear	yes	yes	yes	yes	yes	unclear	unclear	unclear	unclear	yes
Righi	2014	yes	yes	yes	no	yes	yes	yes	yes	yes	unclear	yes	unclear	unclear
Rivera-Chavez	2009	no	yes	yes	yes	yes	yes	yes	yes	yes	unclear	unclear	unclear	yes
Rogina	2014	unclear	yes	yes	yes	yes	yes	yes	yes	yes	unclear	unclear	no	yes
Romualdo	2014	yes	yes	yes	yes	yes	yes	yes	yes	yes	unclear	unclear	unclear	yes
Ruiz-Alvarez	2009	yes	yes	yes	yes	yes	yes	yes	yes	yes	unclear	yes	unclear	yes
Sakr	2008	yes00%	yes	yes	yes	yes	yes	yes	yes	yes	unclear	unclear	unclear	unclear
Scherpereel	2006	unclear	unclear	yes	yes	yes	yes	yes	yes	yes	unclear	unclear	unclear	yes
Schulte	2011	yes	yes	yes	yes	yes	yes	yes	yes	yes	unclear	yes	no	no
Selberg	2000	no	yes	yes	yes	yes	yes	yes	yes	yes	unclear	unclear	unclear	unclear
Seok	2012	no	yes	yes	yes	yes	yes	yes	yes	yes	unclear	unclear	unclear	yes
Shozushima	2011	yes	yes	yes	yes	yes	yes	yes	yes	yes	unclear	unclear	unclear	yes

Sierra	2004	yes	yes	yes	yes	yes	yes	yes	yes	yes	unclear	unclear	unclear	unclear
Su	2012	yes	yes	yes	yes	yes	yes	yes	yes	yes	unclear	yes	unclear	yes
Su	2013	yes	yes	yes	yes	yes	yes	yes	yes	yes	yes	unclear	unclear	unclear
Sungurtekin	2006	unclear	unclear	yes	yes	yes	yes	yes	yes	yes	yes	yes	unclear	yes
Suprin	2000	yes	yes	yes	yes	yes	yes	yes	yes	yes	unclear	unclear	unclear	yes
Takahashi	2014	yes	yes	yes	yes	yes	yes	yes	yes	yes	unclear	unclear	yes	unclear
Talebi-Taher	2014	unclear	yes	yes	yes	yes	yes	yes	yes	yes	unclear	unclear	no	yes
Tan	2016	no	yes	yes	yes	yes	yes	yes	yes	yes	no	no	yes	yes
Tian	2014	no	yes	yes	yes	yes	yes	yes	yes	yes	unclear	unclear	unclear	unclear
Tromp	2012	no	yes	yes	yes	yes	yes	yes	yes	yes	unclear	unclear	unclear	yes
Tsalik	2012	unclear	yes	yes	yes	yes	yes	yes	yes	yes	unclear	yes	no	yes
Tsangaris	2009	yes	yes	yes	yes	yes	yes	yes	yes	yes	unclear	yes	unclear	yes
Tugrul	2002	yes	yes	yes	yes	yes	yes	yes	yes	yes	unclear	unclear	unclear	no
Ulla	2013	yes	yes	yes	yes	yes	yes	yes	yes	yes	yes	unclear	unclear	yes
Vaschetto	2008	unclear	unclear	yes	yes	yes	yes	yes	yes	yes	yes	yes	unclear	yes
Vodnik	2013	no	yes	yes	yes	yes	yes	yes	yes	yes	unclear	unclear	unclear	unclear
Wang	2012	yes	yes	yes	yes	yes	yes	yes	yes	yes	unclear	unclear	unclear	yes
Wang	2013	no	unclear	unclear	unclear	yes	yes	yes	yes	yes	unclear	unclear	unclear	unclear
Wanner	2000	no	yes	yes	yes	yes	yes	yes	yes	yes	unclear	unclear	unclear	unclear
Xiao	2015	yes	yes	yes	yes	yes	yes	yes	yes	yes	unclear	yes	unclear	yes
Yousef	2010	yes	yes	yes	yes	yes	yes	yes	yes	yes	unclear	unclear	unclear	yes
