# Supplementary figures and images for: Biomarkers for diagnosis of sepsis in patients with systemic inflammatory response syndrome: a systematic review and meta-analysis
Source: Springerplus. 2016 Dec 12;5(1):2091. doi: 10.1186/s40064-016-3591-5 (PMC5153391; doi:10.1186/s40064-016-3591-5)

# S3. Forest plots of biomarkers for diagnosis of sepsis

## PCT

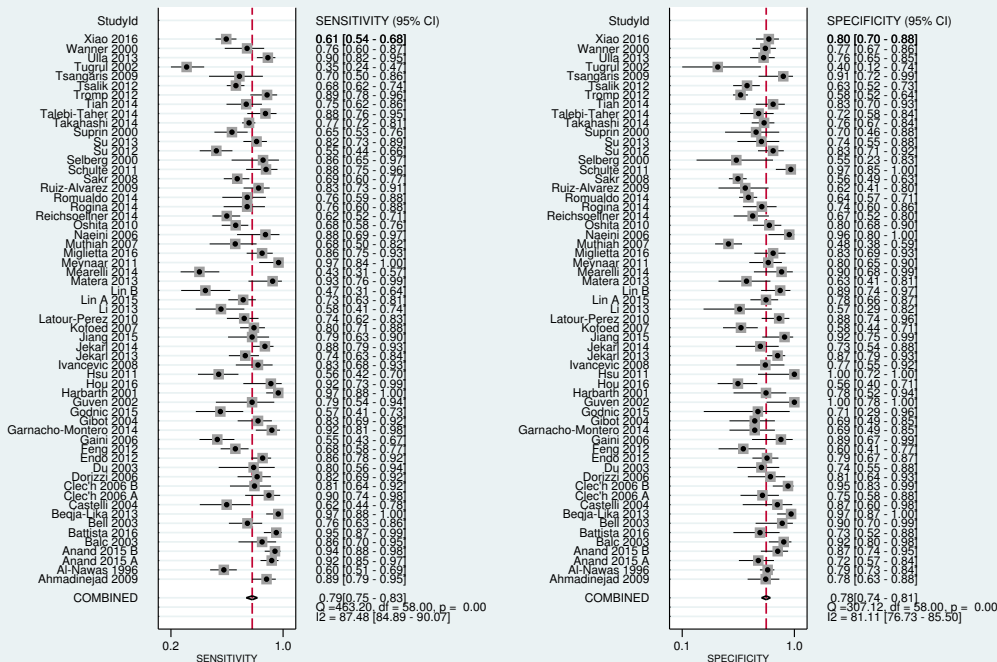

# CRP

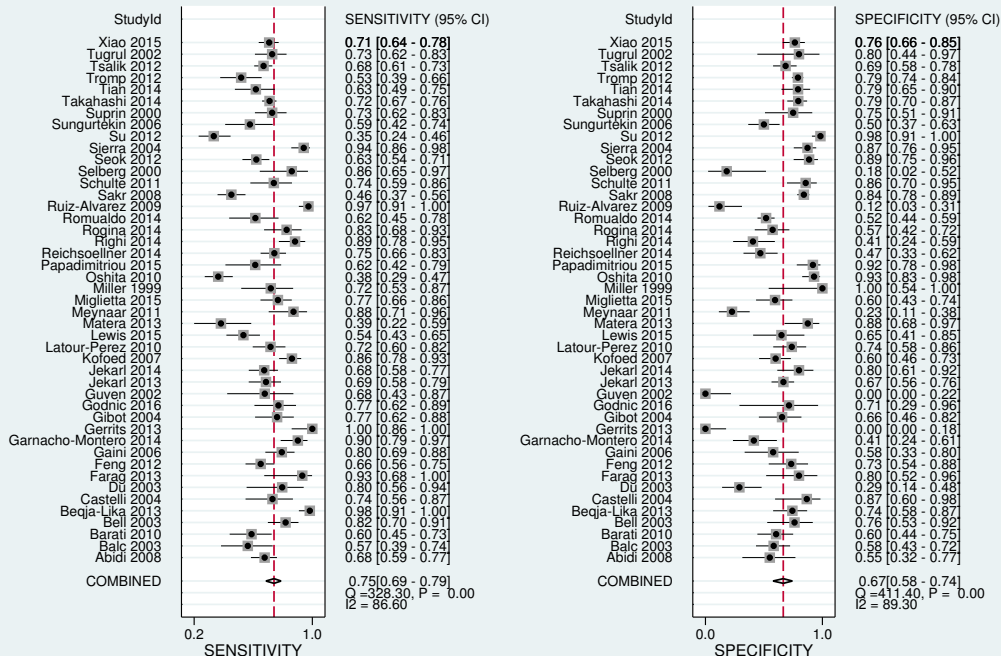

## IL-6

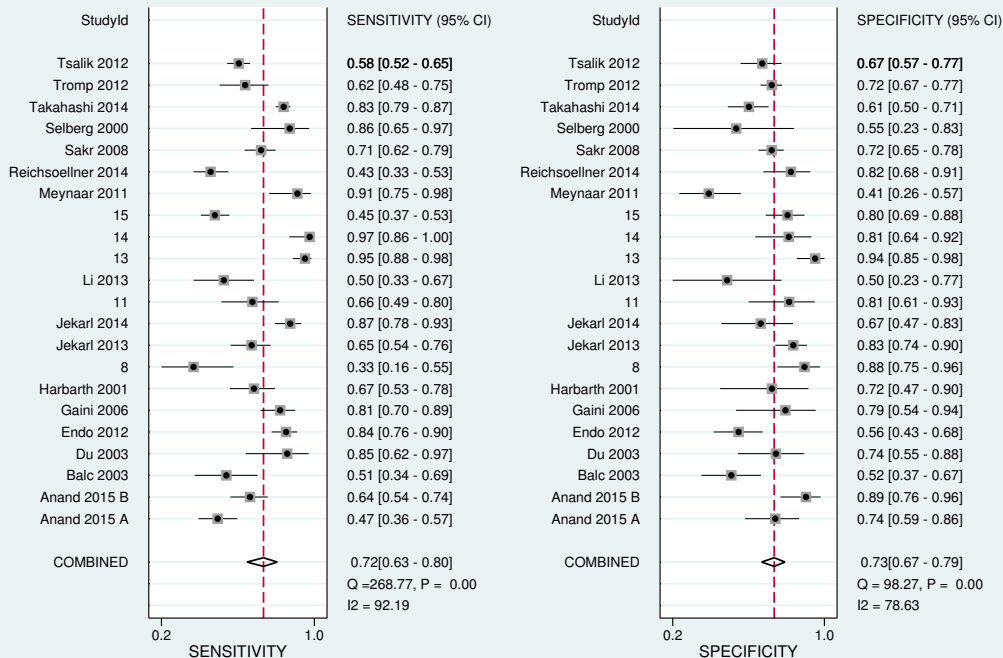

# Presepsin

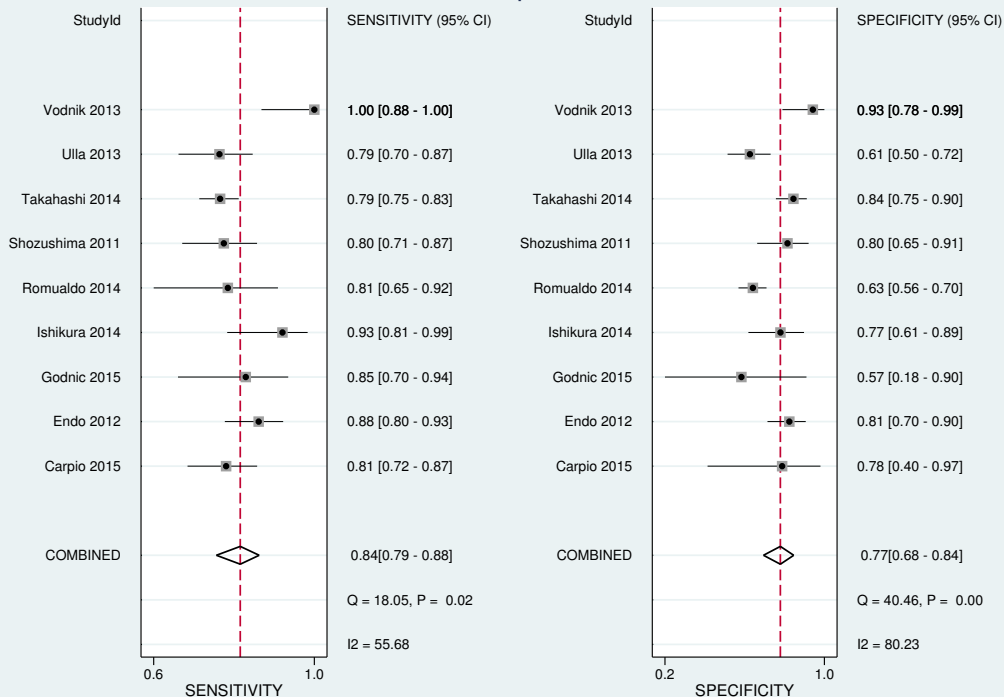

# stREM-1

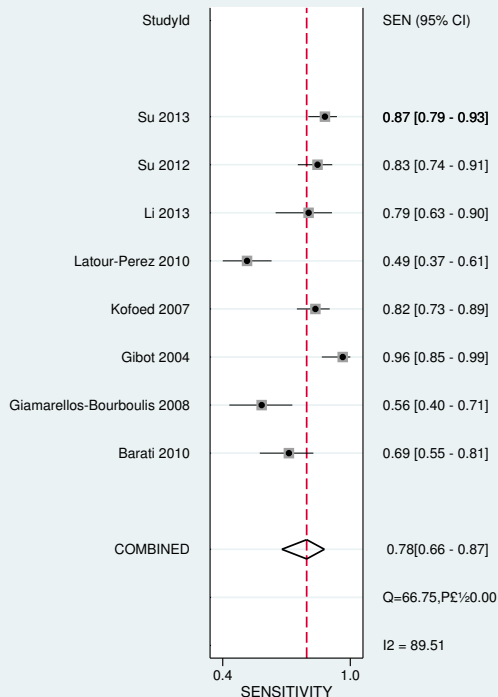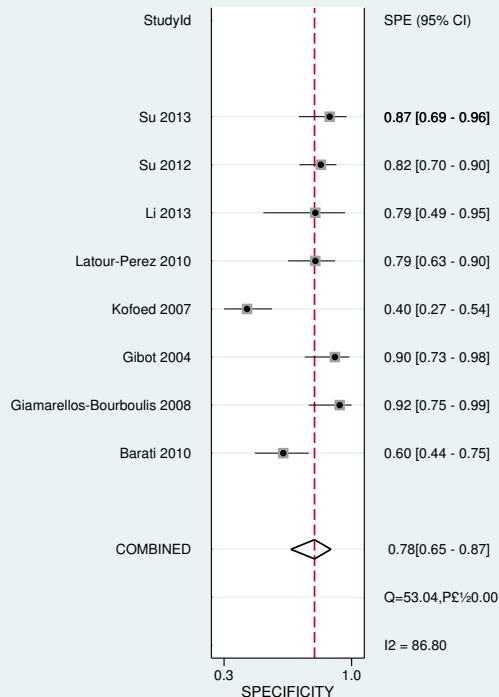

# LBP

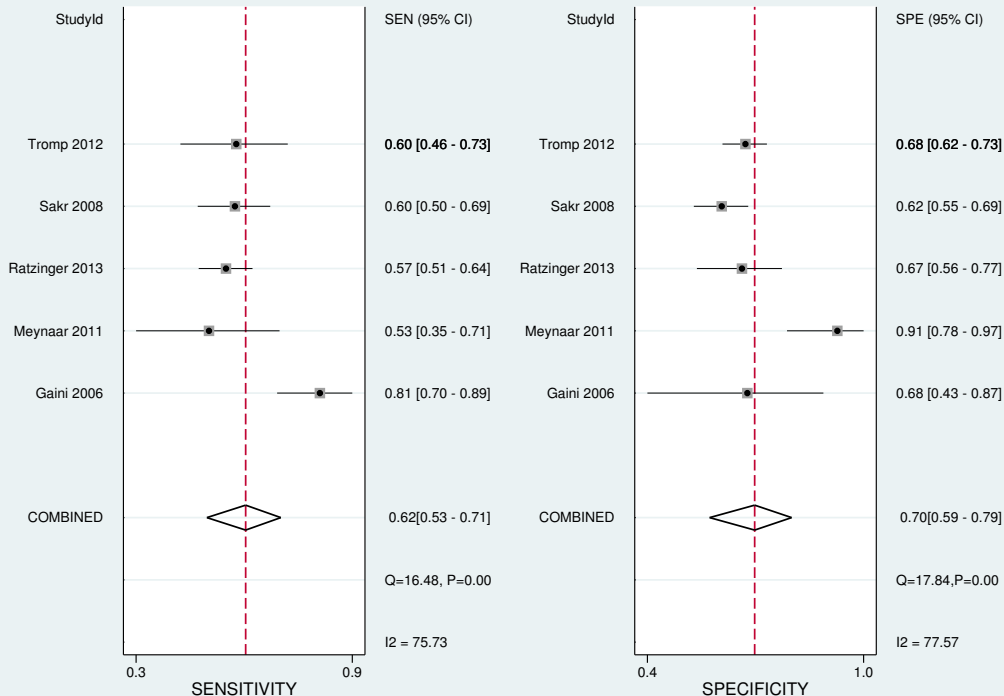

# CD64

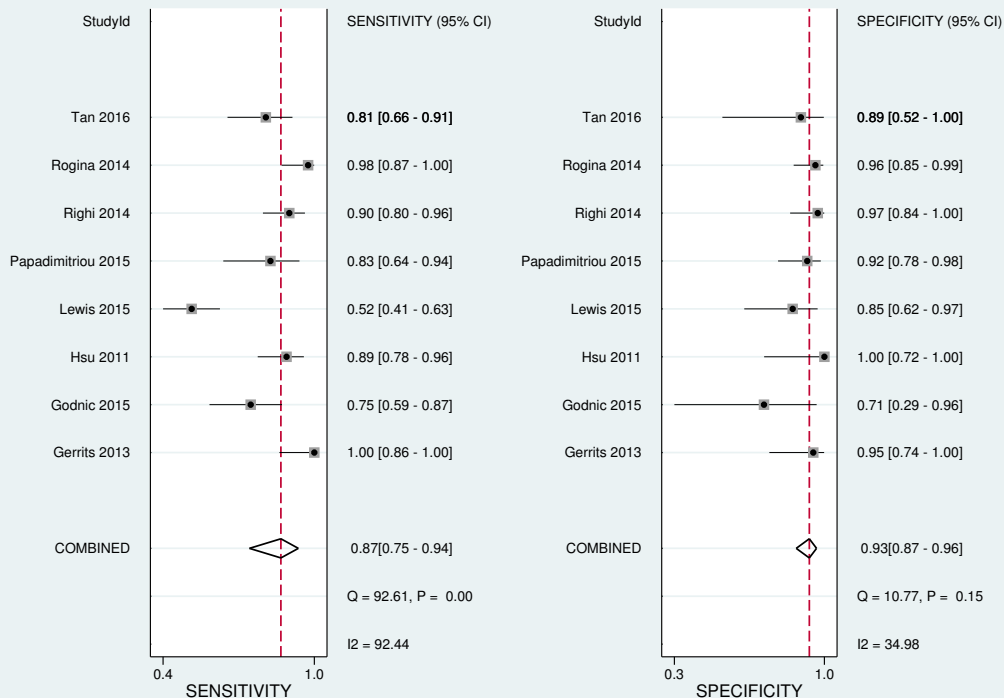

Supplement: Supplementary file 3 — Additional file 3. Forest plots of biomarkers for the diagnosis of sepsis. [file 40064_2016_3591_MOESM3_ESM.pdf]
